# Factors Associated with Maternal Deaths in a Hard-To-Reach Marginalized Rural Community of Bangladesh: A Cross-Sectional Study

**DOI:** 10.3390/ijerph17041184

**Published:** 2020-02-13

**Authors:** Animesh Biswas, Abdul Halim, Abu Sayeed Md. Abdullah, Fazlur Rahman, Sathyanaraynan Doraiswamy

**Affiliations:** 1 Centre for Injury Prevention and Research, Bangladesh (CIPRB), Dhaka 1206, Bangladesh; halim.ogsb@gmail.com (A.H.); sayeedvetmicro@gmail.com (A.S.M.A.); fazlur@ciprb.org (F.R.); 2 United Nation Population Fund (UNFPA), Dhaka 1207, Bangladesh

**Keywords:** maternal death, death review, verbal autopsy, tea garden, marginalized community, Bangladesh

## Abstract

Every year in Bangladesh, approximately 5200 mothers die (172 maternal deaths/100,000 live births) due to maternal complications. The death rate is much higher in hard-to-reach areas and underprivileged communities, such as Bangladesh’s tea gardens. The women living in the tea garden areas are deprived of quality health care services due to inadequate knowledge, education, and access to health care services. Poverty and early marriage, followed by early pregnancy, are also triggering factors of maternal deaths in this community. This study explored the factors associated with maternal deaths in the underprivileged tea garden community in the Moulvibazar district of Bangladesh. It was a cross-sectional study conducted between January and March 2018. All maternal deaths reported by government health care providers in two sub-districts of Moulvibazar during 2017 were selected for community verbal autopsy using a structured questionnaire. Descriptive analysis was performed on quantitative data, and content analysis was performed on qualitative data. A total of 34 maternal deaths were reported in the two sub-districts in 2017, among which 15 deaths (44%) occurred in the tea garden catchment areas, where about 34% people live in the two upazilas. The majority of the mothers who died in the tea gardens delivered their babies at home (80%), many of whom also died at home (40%). Only 27% of women who died in the tea gardens received four or more antenatal care visits. Post-partum hemorrhage was found to be the leading cause of death (47%), followed by anemia (33%) and eclampsia (20%). There is a persistent high maternal mortality observed in the marginalized tea gardens, as compared to the general community of the Moulvibazar district, Bangladesh. The sustainable development goal (SDG) that has been set for maternal mortality rate (MMR) is 70/100,000 live births in Bangladesh. The findings of our study show that focused intervention is needed to reduce the burden of maternal deaths, which will improve the overall maternal health situation and also reach the SDG on time.

## 1. Introduction

Maternal death is a global public health agenda, costing more than 3 million women their lives every year [1]. Globally, every day approximately 830 women die from pregnancy and childbirth-related complications. The maternal mortality ratio (MMR) is 211 (i.e., 211 deaths per 100,000 live births), 99% of which occur in developing countries [2]. Bangladesh has achieved remarkable success in reducing maternal deaths in the last two decades [3]. However, there is still a long way to go to reach the sustainable development goal (SDG) target, which is 70 or fewer maternal deaths per 100,000 live births. The estimated maternal mortality in 2017 was 172 per 100,000 live births in comparison to 194 per 100,000 live births in 2010 [4,5]. The annual reduction rate is 5.6% at present. Though facility delivery increased from 9.1% to 28.8%, home delivery was still more than 62% [6]. The government of Bangladesh has prioritized the reduction of maternal death and in 2010 introduced the Maternal and Perinatal Death Review (MPDR) system in one district, which has now expanded to cover ten districts and approximately 20 million people [7]. Through the MPDR system, every maternal death has been captured and reported to the MPDR focal point by government health and family planning grassroots-level workers. Subsequently, community verbal autopsies have been conducted for every death at the residence of the deceased by first-line field supervisors, from the health and family planning department, in order to ascertain the possible causes of death [8,9]. The results of the verbal autopsy have been reviewed by the death review committee, led by the health managers [10], and the data analyzed by gynecologists and obstetricians in the medical college hospital at the divisional level [10,11]. The tea garden community, with a population of 400,000 is in the eastern part of the country, which is marginalized and hard to reach. The recent government data has shown that 39% of total maternal deaths in the Moulvibazar district are from the tea garden community. The district has a population of approximately two million and approximately 92 tea gardens. About 300,000 people are from the tea garden community. This study explored the factors associated with maternal deaths that have occurred in the underprivileged tea garden community and drawn comparisons with the corresponding general community in the Moulvibazar district of Bangladesh.

## 2. Materials and Methods

The study was conducted using the cross-sectional method. The duration of the study was from January to March 2018. The verbal autopsies of maternal deaths in two sub-districts of Moulvibazar in 2017, which were conducted by the government health care providers at community level using a structured questionnaire, were reviewed for this study. In-depth interviews were conducted with the deceased’s family members from in the tea gardens communities.

### 2.1. Study Site

The study was conducted in two purposively selected upazilas (sub-districts) of the Moulvibazar district. The maternal deaths reported and reviewed in the study areas in the year 2017 by the existing health system and the MPDSR program were considered in this study. The study covered 507,880 people in two sub-districts, or upazilas, of the Moulvibazar district: Sreemangal (278,232 people) and Kamalganj (229,648 people) [12]. The tea garden population living in these two upazilas is 171,075 [13,14,15].

### 2.2. Data Collection Tools

Maternal and Perinatal Death Surveillance and Responses (MPDSR) is a national program of the Ministry of Health and Family Welfare (MoH & FW) which follows a national guideline for MPDSR, that includes a training manual and data collection tools. The verbal autopsy tool is adopted from the World Health Organization (WHO) tool and contextualized according to country context. The study used the exiting MPDSR community death notification and verbal autopsy tools to gather quantitative data. The community death notification slip was used for the notification of maternal deaths in the community. The tool contains some key variables including the deceased’s name, age, time and place of delivery, place of death and detailed address. The verbal autopsy tool was used to collect in-depth information on maternal death at the household level. The verbal autopsy tool is a semi-structured questionnaire with several sections include questions on general information, antenatal care, delivery care, postnatal care, pregnancy-related complications, previous history and health-seeking behavior. For qualitative data, a guideline was developed for an in-depth understanding of the causes of death.

### 2.3. Training of the Field-Level Health Workers

The Bangladeshi government provided training for the different tier health care providers, using the MPDSR national training manual and tools at the sub-district level. The field-level government health care providers received training on community death notification, and the first-line supervisors on the field-level received training in community verbal autopsy. The training focused on the procedure to gather information on deaths from the community, reporting, registering deaths, and conducting and reporting verbal autopsies at the household level.

### 2.4. Death Notification

Trained grassroots-level health care providers, including health assistants (HA) and family welfare assistants (FWA), used a community network to identify and record a maternal death from their working catchment area. The community networks included community volunteers, members from a community group and community support group, teachers, religious leaders, members of local government, medicine shopkeepers, traditional birth attendants, village doctors, and other community members. After gathering initial information, the health care providers visited households to confirm suspected maternal deaths following the operational definition used in the MPDSR guidelines. The HA or FWA filled out the death notification slip and reported back to the nearby community clinic, which was the lowest-level primary health care center assigned per 6000 people, and also reported to the MPDSR focal person of the upazila.

### 2.5. Verbal Autopsy

The first line field supervisors, including health inspector, assistant health inspector and family planning inspector, were assigned to conduct the verbal autopsy of the maternal death at the household level. The health care provider was appointed by the MPDSR focal person of the upazila when they received a maternal death notification slip from a field-level health care provider. The health worker visited the household, met the relatives, and determined which respondent to interview. The health care provider used a structured community verbal autopsy form for the face-to-face interview, if needed, and also received information from associate respondents such as family members and neighbors who could provide more details. The health care provider acquired informed consent before the interview and returned the completed verbal autopsy form to the MPDSR focal person at the upazila.

### 2.6. In-Depth Case Studies

A guideline was developed to collect comprehensive information on maternal deaths. One trained research assistant was assigned to visit each of the deceased’s households, to meet with the family members and listen to each of the case stories. The information focused on the exploration of social behaviors, barriers, gaps, practices and challenges that may lead to the death of a woman.

### 2.7. Monitoring and Quality Assurance

MPDSR’s monitoring and quality assurance system monitors both community death slip notifications and verbal autopsy data collection. MPDSR’s quality improvement (QI) committee functions at the sub-district level, where the sub-district focal reports to the QI committee chair. Every monthly coordination meeting of the health and family planning department also discussed and analyzed the monthly MPDSR data, where all field-level health care providers participated. 

Moreover, monthly coordination meetings were held at the upazila health complexes (primary health care center) where all field-level health workers participated to share their monthly updates. The MPDSR focal person and the chair of the MPDSR QI committee provided feedback on the data obtained. Among all the cases, 10% of the case studies were checked to ensure quality and consistency with the verbal autopsy data.

### 2.8. Cause Assignment of Verbal Autopsy

The verbal autopsy forms were reviewed by the physicians (consultant Obs-Gyane) who were trained earlier on this to assign the causes of death. If any query arose, the physicians discussed in a team to determine the causes of the deaths. Verbal autopsy data with inadequate information were coded as ‘undetermined’.

### 2.9. Data Analysis

The data analyst entered all of the data in Excel, cleaned it, and transferred it to SPSS version 21.0 for descriptive analysis. Data from tea garden and non-tea garden groups were compared using *t*-tests to determine whether differences between them were statistically significant or not. For qualitative data, content analysis was performed. The case reports were reviewed, read, and re-read to identify social determinants of maternal death and presented in a table by an anthropologist.

### 2.10. Ethical Approval

Ethical approval was obtained from the Ethical Review Committee, Centre for Injury Prevention and Research Bangladesh (ERC/CIPRB/2016/10). Informed consent was acquired from each respondent before the interview. The health workers maintained the anonymity and confidentiality of respondents through the process, taking a non-blaming approach. Participation in each interview was voluntary, and respondents were informed that they could skip any questions or leave the discussion at any point.

## 3. Results

### 3.1. Maternal Death Data Captured and Reported

**Figure 1:**
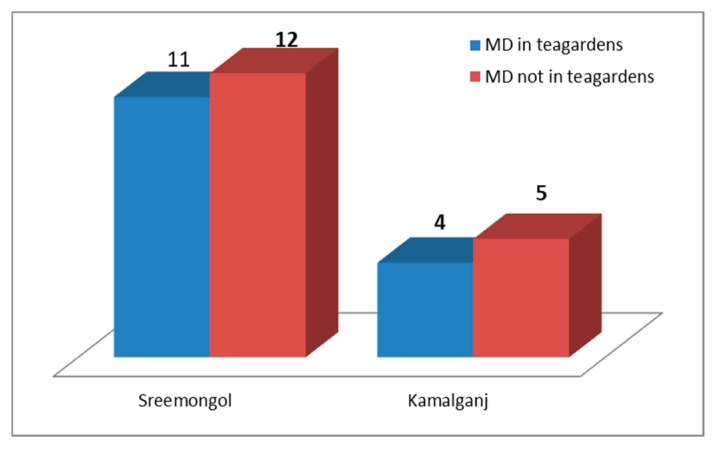
Comparing the maternal deaths in the tea gardens and outside of the tea garden communities in the sub-districts.

A total of 34 maternal deaths were identified and reported in the two sub-districts (Sreemangal and Kamalganj) of the Moulvibazar district in 2017. Among those, 15 deaths occurred in the tea garden catchment area. In Sreemangal, approximately 48% of deaths occurred in the tea garden, whereas 44% occurred in the Kamalganj upazila. The data were statistically significant (*p* value 0.001012, CI 95%) (Figure 1). Rajghat union had the highest number of maternal deaths out of the other unions in the Sreemangal upazila (*n* = 7) (Table 1).

### 3.2. Demographic Characteristics of the Deceased

The mean age of mothers who died inside the tea gardens was lower (24.9 y) than the age of those who died outside of the tea gardens (30.3 y). All of the women who died in the tea gardens had an education level below primary level. Around 27% of the women in the tea gardens received four or more antenatal care visits in comparison to outside of the tea garden, which is approximately 34%, with *p* value 0.004296, CI 95% (Table 2).

### 3.3. Place of Death of Women

**Figure 2:**
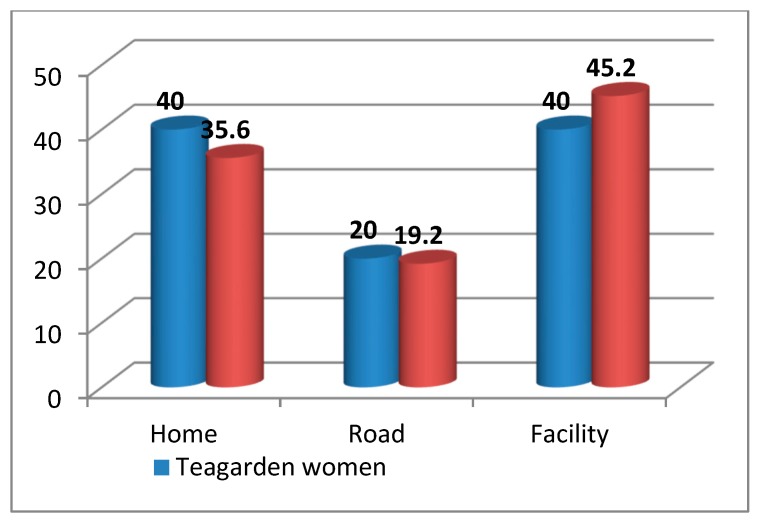
Comparing place of maternal deaths among tea garden and general communities.

The tea garden mothers more often died in their homes (40%) and less frequently in health facilities (40%), as compared to mothers outside of the tea garden who died at home (35.6%) or in a facility (45.2%), which is statistically significant (*p* value < 0.0000001, CI 95%) (Figure 2).

### 3.4. Place of Delivery and Birth Attendant during Delivery

A total of 80% of the tea garden mothers delivered at home, whereas 58.5% of mothers outside of the tea gardens delivered at home, which is statistically significant (*p* value < 0.0000001, CI 95%). Every birth in the tea gardens was performed by traditional birth attendants. A total of 55.4% of mothers outside of the tea gardens delivered at home by means of traditional birth attendants.

### 3.5. Causes of Maternal Deaths

**Figure 3:**
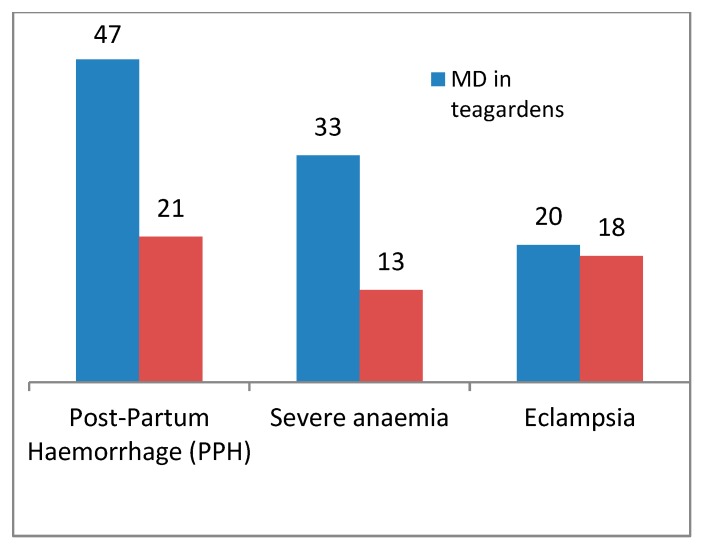
Cause of maternal deaths (*n* = 35) in tea gardens and outside tea gardens.

In the selected two sub-districts, 40% of all maternal deaths occurred due to post-partum hemorrhage, which is the leading cause of maternal deaths. The second leading cause was eclampsia (23%). Whereas among the 15 maternal deaths in the tea gardens, 47% occurred due to post-partum hemorrhage, which is statistically significant (*p* value < 0.0000001, CI 95%), 33% occurred due to severe anemia, and 20% occurred due to eclampsia (Figure 3).

### 3.6. Decision and Transportation Delay among the Deaths in Tea Gardens

Among the 15 maternal deaths in the tea gardens, in 10 (67%) cases, it took one to two hours to make decisions after complications arise. Five (33%) cases needed around 2 h to reach the facility. Four mothers (27%) were not able to reach the facility and died in transit to the facility (Table 3).

### 3.7. Findings from the Case Studies of Maternal Deaths in the Tea Gardens

The in-depth results of the cases in the tea gardens included age, profession, place of delivery and deaths, community delay, complications and causes of deaths. Social barriers of these deaths are also focused on in this table. The deaths occurred at an early age for the women. Lack of knowledge and practices regarding seeking maternal care, birth planning, delivery at the facility by skilled providers, ignorance of receiving care during pregnancy, during delivery and after delivery, were all typical features. The delay in making decisions after complications and also transportation delay was found to be common among the tea garden women (Table 4).

## 4. Discussion

The key findings of the study include that, of the maternal deaths in tea garden areas, 47% occurred due to post-partum hemorrhage (PPH), 33% occurred due to anemia, and 20% occurred due to eclampsia. PPH is also the leading cause of maternal death in the general communities (40%). The community delay includes decision delay and treatment delay, which were found to be common in tea garden areas. The main social factors behind the deaths in the tea gardens include the ignorance of receiving maternal care, ignorance in birth planning, and dependency on the traditional birth attendants during delivery and maternal complication.

The tea garden constitutes only one-third of the total population in these two sub-districts, but almost half of all maternal deaths occurred here. We found that, out of 34 maternal deaths in the two sub-districts, 15 deaths occurred in the tea garden catchment areas in 2017. The proportion is slightly higher than the report of the Directorate General of Health Services (DGHS) in 2014, that out of 120 maternal deaths, 47 deaths occurred in the tea garden area of the Moulvibazar district of Bangladesh. To accelerate the process, the government has introduced midwifery courses and allocation of the post to perform midwifery care in Bangladesh [16].

In this study, due to the low number of maternal deaths, we did not calculate the ratio; however, the recent Bangladesh maternal mortality and health care survey (BMSS) in 2016 mentioned that the current maternal mortality ratio in Bangladesh has stalled, in comparison to the last survey in 2010 [17]. As the tea garden is one of the hardest to reach areas with marginalized communities, this might play a role in the considerable number of maternal deaths [18].

Among all of the maternal deaths found in the two above mentioned sub-districts, post-partum hemorrhage (PPH) was responsible for 40% cases, and 23% occurred due to eclampsia. The scenario is almost the same in the tea garden, where 47% of mothers died from PPH and 20% from eclampsia. However, anemia was found to be another significant cause there, resulting in 33% of total maternal death. BMSS in 2016 reported that 55% of maternal deaths occurred due to PPH and eclampsia [17].

This study also revealed that 80% of the tea garden deceased mothers delivered at home, which is much higher compared to mothers outside of the tea gardens (58.5%), as well as the whole country, where the rate is 53% according to the BMMS 2016 report [17]. Since the majority of the deliveries in the tea gardens were conducted by the traditional birth attendant, future initiatives should focus on increasing the number of births attended by a skilled birthing attendant [19,20].

While analyzing the qualitative data, some social factors of maternal deaths in the tea gardens were revealed through the in-depth interviews, which included ignorance, negligence, and lack of knowledge and practices of maternity care. Believing in myths and superstition were also found to be indirectly responsible in the case of one maternal death. Moreover, a lack of coordination in the case of providing maternal and neonatal health care and the improper referral for related complications was also responsible, which was mentioned in a study done earlier in the tea garden [19].

The delay in decision making and transportation during maternal complications were found common within tea gardens in this study, which is quite similar to another study where community delay works as a vital factor in maternal deaths. In the majority of cases, it was found that the delivery was done by traditional birth attendants (TBA) who are very influential in the tea gardens, which constituted an essential factor in community delay [21].

Although the deaths were reported in the tea gardens, there was some delay in notification. Therefore, in some of the cases, verbal autopsy was not conducted within 21 days, as suggested in the national guideline. This may have resulted in an increased chance of recall bias. Moreover, the number of deaths is limited to tea gardens located in two sub-districts of the Moulvibazar district, which may not reflect the whole tea garden population, due to small sample size.

A considerable gap also existed in the timely referral of women from tea garden to the referral center, which should be minimized, because another study in the tea garden suggested that appropriate referral can save many maternal lives [20].

## 5. Conclusions

Marginalized communities such as tea gardens are still living without access to quality maternal health care. The high number of maternal deaths demand special intervention addressing the disparity in maternal health. Bangladesh is well on track to achieving the sustainable development goal regarding maternal deaths; however, the findings clearly highlight that improvement of quality maternal health care services in this community could affect overall maternal death reduction in the district. Bagan Mayer Jonno is an intervention which started to work for tea garden women in selective tea gardens with the collaboration of Government of Bangladesh (GoB), tea garden authorities, United Nations Population Fund (UNFPA) and Centre for Injury Prevension and Research, Bangladesh (CIPRB). Further investigations may be required on a larger scale in similar types of marginalized community to explore similar types of challenges for better intervention package design.

## Figures and Tables

**Table 1:** Number of deaths in the tea gardens in 2017.

Upazila	Union	No. of Tea Gardens	No. of Maternal Deaths
Sreemangal	Rajghat	4	7
	Sathgaon	1	1
	Kalighat	1	1
	Ashirdrone	1	2
Kamalganj	Islampur	1	1
	Madhabpur	1	1
	Shamshernagar	1	2
Total		10	15

**Table 2:** Demographic characteristics of the deceased mothers.

Characteristics	Total Deaths (*n* = 34)	Mothers Who Died in Tea Gardens (*n* = 15)	Mothers Who Died Outside of the Tea Gardens (*n* = 19)
Median age of mother in years [Inter Quartile Range]	27.6 (18–38)	24.9 (18–30)	30.3 (20–38)
Mother’s education (n [%])			
Below primary level	20 (58.8)	15 (100)	5 (26.3)
Primary education	8 (23.5)	-	8 (42.1)
Secondary education	6 (17.7)	-	6 (31.6)
Maternal characteristics			
Accessed ANC four or more times	11 (32.4)	4 (26.7)	7 (33.8)
Parity (n [%])			
1	8 (23.6)	2 (13.3)	6 (34.0)
2–4	20 (58.8)	10 (66.7)	10 (52.2)
5 or more	6 (17.6)	3 (20.0)	3 (14.8)

**Table 3:** Delay of mothers in decision making and transportation.

Delays	15 Maternal Deaths in the Tea Gardens, *n* (%)
1st delay: Decision to seek care	
Within 1 h	3 (20)
1–2 h	10 (67)
Decided not to go to the facility	2 (13)
2nd delay: Time to transfer	
Within 1 h	1 (7)
1–2 h	5 (33)
2–3 h	3 (20)
Could not transfer	4 (27)
No transport	2 (13)

**Table 4:** Detailed information of the maternal deaths in tea gardens (*n* = 15).

Case	Gestational Period	Complications	Cause of Death	Summary Case Description Obtained from Case Reports
Case 1	36 weeks	Anemia, Edema, vomiting	Severe Anemia	It was her third pregnancy. She did not receive any ANC during her pregnancy. She was suffering from anemia and swelling of face and legs; it was overlooked by her family members. After delivery at home by TBA, the mother had continuous vomiting. Twelve days after birth, the mother died from anemia, edema and vomiting without getting any treatment.
Case 2	42 weeks	Anemia, Edema on face and leg	PPH	The mother could not detect her pregnancy for up to six months. Though she was suffering from severe abdominal pain, she attributed it to hard work. She never received any antenatal care and delivered at home by TBA. Immediately after delivery, PPH started, and she died at home.
Case 3	38 weeks	Eclampsia, Prolonged labor with PPH	PPH	It was her second pregnancy. The mother was suffering from severe headaches due to pre-eclampsia during pregnancy. But all family members ignored these issues. She had labor pain for more than 19 h, but the TBA tried to conduct the delivery in the traditional way. After delivery PPH started and the mother died at home.
Case 4	30 weeks	Anemia, Edema	PPH	It was her third pregnancy, and she had a previous history of miscarriage. She suffered from anemia and preeclampsia. She received two ANC visits at the tea garden hospital. She was advised to deliver at a facility, but her family members decided to conduct the delivery at home by TBA. PPH started immediately after birth, and the mother died at home.
Case 5	31 weeks	Anemia, Edema	Severe anemia	Though it was her third pregnancy, the mother and her family members were unaware of maternal health care. She did not receive any ANC during pregnancy and did not take any essential medicine. Her face and legs became swollen at the fifth month of pregnancy. After delivery at home by TBA, the mother died at home from severe anemia.
Case 6	32 weeks	Edema, Unconsciousness	Severe anemia	It was her fifth pregnancy, and she suffered from swelling of the face and limbs. Though she received one ANC visit during pregnancy, she did not follow the given advice. She delivered at home by TBA. The mother died at home after five hours of delivery.
Case 7	32 weeks	Retention of placenta with PPH	PPH	It was her first pregnancy, and delivery pain started at midnight while she was at home. Her husband called the TBA, who tried and delivered a baby on the morning around 6.30 am. Unfortunately, PPH began to due to the retained placenta. She was carried to Bagan hospital where a paramedic (Bagan Midwife) attempted to treat her. Then at 9 am, she was referred to the tea garden central hospital and later Osmani Medical College Hospital at 11 am, but the mother died on the road at noon.
Case 8	38 weeks	Anemia, Edema on face and leg	Severe anemia	The family members believed myths that delivery should be conducted at home because if a pregnant woman went outside, it would be bad for her pregnancy. After birth at home by TBA, her face and limbs became swollen and her abdomen distended with frothy discharge coming out of her mouth. They then carried the mother to the tea garden hospital. But the paramedic and compounder could not detect the problem. Then they decided to come back home, and the mother died on the road.
Case 9	30 weeks	Severe anemia, Retained placenta, PPH	PPH	The mother was identified as high risk with severe anemia during her first ANC visit at the tea garden hospital by a paramedic (Bagan midwife). She and her husband were counseled on the importance of pregnancy care. She went to her mother’s home at the sixth month of pregnancy and could not receive other ANC. After delivery by TBA, the placenta could not be removed, resulting in severe PPH. Then she was carried to the tea garden hospital where she was referred to UHC. She died on the road on the way to UHC.
Case 10	31 weeks	Prolonged labor with PPH	PPH	It was her first pregnancy. The mother was admitted to UHC with obstructed prolonged labor and delivered a baby by caesarian section. But PPH started immediately after delivery, and then the mother was referred to the district hospital. But the family members decided to admit her in the private hospital in that sub-district. However, this hospital could not manage the complications and referred the mother to Osmani medical hospital. But it took a long time to reach the destination and the mother died just after treatment started at Osmani Medical College Hospital.
Case 11	32 weeks	Anemia, Edema	PPH	She was suffering from severe headaches. For this complaint, her husband carried her to a traditional healer (Kabiraj) where conventional treatment was provided. After seeking treatment, the mother came to her home but suddenly fell in the yard. Convulsions started and after a while she fell unconscious. Then she was carried to the tea garden hospital, followed by the district hospital and Osmani Medical College Hospital. But she died after the last admission.
Case 12	28 weeks	Severe headache	Eclampsia	Delivery occurred at home by TBA. The TBA explained that it was challenging to remove the placenta and hemorrhage started during the removal of the placenta with rough handling. After delivery, the mother suffered from anemia, swelling of legs and face. After six days, the mother was referred to UHC from her home by a paramedic, then referred to the District Sadar hospital and then to Osmani Sadar Hospital but the mother died before reaching that facility.
Case 13	30 weeks	Twin pregnancy	Eclampsia	It was her fourth pregnancy. The last three deliveries occurred at home by TBA, and two of them were stillbirths. Though she felt a headache and abdominal pain, she did not have treatment during this pregnancy. At the time of delivery, she felt severe pain in the abdomen. She did not even know that she was carrying twins. She was taken to the tea garden hospital and then to the district hospital. But immediately after reaching the hospital, the mother died, with convulsions.
Case 14	29 weeks	Anemia, Edema	PPH	It was her third pregnancy. The mother was suffering from pre-eclampsia and severe anemia during pregnancy. She also suffered from hemorrhoids. Her family members ignored special care during pregnancy. They admitted her into hospital with complications. They delayed in seeking treatment, and the mother died after delivery due to PPH.
Case 15	30 weeks	Anemia, Abdominal pain, High blood pressure	Severe anemia	It was her fourth pregnancy, and she had a history of miscarriage. She had a symptom of pre-eclampsia (decided by a physician) during pregnancy, but her family members did not take this issue seriously. The mother delivered at home by TBA. PPH started immediately after delivery. Then the mother was carried to Bagan Hospital, later referred to UHC, and finally transferred to the district hospital, and the mother died there.

## Abbreviations

FWA	family welfare assistant
HA	health assistants
MPDR	Maternal and Perinatal Death Review
MPDSR	Maternal and Perinatal Death Surveillance and Responses
MoH and FW	Ministry of Health and Family Welfare
PPH	post-partum hemorrhage
QI	quality improvement
SDG	sustainable development goal
TBA	Traditional birth attendant
